# HER2 Heterogeneity Is Associated with Poor Survival in HER2-Positive Breast Cancer

**DOI:** 10.3390/ijms19082158

**Published:** 2018-07-24

**Authors:** Mari Hosonaga, Yoshimi Arima, Oltea Sampetrean, Daisuke Komura, Ikuko Koya, Takashi Sasaki, Eiichi Sato, Hideyuki Okano, Jun Kudoh, Shumpei Ishikawa, Hideyuki Saya, Takashi Ishikawa

**Affiliations:** 1 Department of Breast Surgery and Oncology, Tokyo Medical University, 6-7-1 Nishishinjuku, Shinjuku-ku, Tokyo 160-0023, Japan; tishik@gmail.com; 2 Division of Gene Regulation, Institute for Advanced Medical Research, Keio University School of Medicine, 35 Shinanomachi, Shinjuku-ku, Tokyo 160-8582, Japan; arima@z7.keio.jp (Y.A.); oltea@a6.keio.jp (O.S.); hsaya@a5.keio.jp (H.S.); 3 Department of Genomic Pathology, Medical Research Institute, Tokyo Medical and Dental University, 1-5-45 Yushima, Bunkyo-ku, Tokyo 113-8510, Japan; komura-daisuke@umin.ac.jp (D.K.); sish.gpat@mri.tmd.ac.jp (S.I.); 4 Department of Physiology, Keio University School of Medicine, 35 Shinanomachi, Shinjuku-ku, Tokyo 160-8582, Japan; koya-ikuko@a5.keio.jp (I.K.); hidokano@a2.keio.jp (H.O.); 5 Center for Supercentenarian Medical Research, Keio University School of Medicine, 35 Shinanomachi, Shinjuku-ku, Tokyo 160-8582, Japan; sasasa@z5.keio.jp; 6 Department of Pathology (Medical Research Center), Institute of Medical Science, Tokyo Medical University, 6-7-1 Nishishinjuku, Shinjuku-ku, Tokyo 160-0023, Japan; sato-e@tokyo-med.ac.jp; 7 Laboratory of Gene Medicine, Keio University School of Medicine, 35 Shinanomachi, Shinjuku-ku, Tokyo 160-8582, Japan; jkudoh@dmb.med.keio.ac.jp

**Keywords:** HER2, heterogeneity, breast cancer, poor prognosis, caspase-1

## Abstract

Intratumoral human epidermal growth factor receptor 2 (HER2) heterogeneity has been reported in 16–36% of HER2-positive breast cancer and its clinical impact is under discussion. We examined the biological effects of HER2-heterogeneity on mouse models and analyzed metastatic brains by RNA sequence analysis. A metastatic mouse model was developed using 231-Luc (triple negative cells) and 2 HER2-positive cell lines, namely, HER2-60 and HER2-90 which showed heterogeneous and monotonous HER2 expressions, respectively. Metastatic lesions developed in 3 weeks in all the mice injected with HER2-60 cells, and in 69% of the mice injected with HER2-90 and 87.5% of the mice injected with 231-Luc. The median survival days of mice injected with 231-Luc, HER2-60, and HER2-90 cells were 29 (*n* = 24), 24 (*n* = 22) and 30 (*n* = 13) days, respectively. RNA sequence analysis showed that *CASP-1* and its related genes were significantly downregulated in metastatic brain tumors with HER2-60 cells. The low expression of caspase-1 could be a new prognostic biomarker for early relapse in HER2-positive breast cancer.

## 1. Introduction

Genetic heterogeneity within the same tumor has been demonstrated by exome sequencing in primary renal cell cancer. Gene expression signatures of good and poor prognoses have been detected in the same tumor in different regions [1]. Both genetic heterogeneity and nongenetic heterogeneity are usually present [2,3], which may cause a difference in the therapeutic response and biological behavior.

In a previous study of mutational evolution in a lobular breast tumor profiled at single nucleotide resolution, the number of mutations increased more in the metastatic site than in the primary breast cancer in patient-matched samples [4]. The degree of intratumoral heterogeneity including genetic and nongenetic alterations was associated with the progression of in situ human breast cancer to an invasive phenotype [5]. Thus, intratumoral heterogeneity was associated with the progression and metastasis of breast cancer.

Intratumoral heterogeneity of HER2 expression and gene amplification has been reported in 16–36% of patients with HER2-positive breast cancer [6,7]. The clinical impact of intratumoral HER2 heterogeneity is currently under discussion. Patients with HER2 genetic heterogeneity according to the American Society of Clinical Oncology/College of American Pathologist (ASCO/CAP) guidelines have been reported to have shorter survival times than patients without HER2 genetic heterogeneity [6]. However, another study demonstrated that the proportion of tumor cells with HER2/centromere on chromosome 17 (CEP17) ratios of more than 2.2 was not correlated with a poor prognosis [8]. Both of these results were from studies involving treatment without trastuzumab. Importantly, it is also necessary to examine these findings in cases treated with standard treatments including trastuzumab.

In our previous study, we generated 2 HER2-positive cell lines from a triple negative cell line of MDA-MB 231 cells (231-Luc), namely, HER2-60 and HER2-90, by introducing wild-type *HER2* and collected HER2-overexpressed cells. HER2-60 cells consist of 63.2% HER2-overexpressed cells and 36.8% HER2-negative cells, whereas HER2-90 cells consist mostly of HER2-overexpressed cells [9]. Thus, the HER2-60 cell line represents a heterogeneous type, whereas the HER2-90 cell line represents a monotonous type.

In the present study, we used these cell lines to examine whether HER2 heterogeneity affects the malignant potential of breast cancer in vivo. Our model has an advantage of analyzing the interaction of cancer cells, which originated from breast cancer patients, and stromal cells, which originated from mice. We investigated the metastatic brains of each mice injected with 231-Luc, HER2-60, or HER2-90 cells. According to the heterogeneity of HER2 expression, we also examined the prognosis of HER2-positive breast cancer patients, who were treated by neoadjuvant chemotherapy with trastuzumab.

## 2. Results

### 2.1. Mouse Model

#### 2.1.1. Heterogeneous HER2 Expression Is Associated with Short Survival in Metastatic Breast Cancer

**Figure 1:**
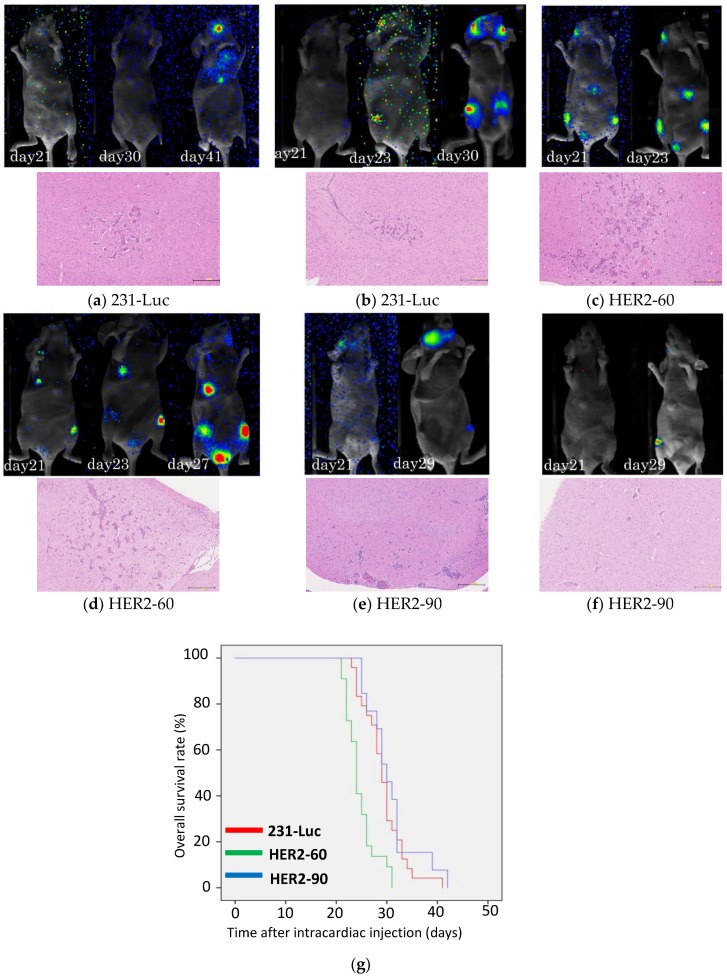
Heterogeneous HER2 expression is associated with a short survival time in metastatic breast cancer. (**a**–**f**) Representative bioluminescence images of mice after intracardiac injection (**upper panel**). H&E staining of formalin-fixed, paraffin-embedded metastasized brain sections. Scale bar, 250 μm (**lower panel**). (**a**,**b**) Injected with 231-Luc cells; (**c**,**d**) injected with HER2-60 cells; (**e**,**f**) injected with HER2-90 cells; (**g**) survival curves of mice after intracardiac injection of 231-Luc (*n* = 24), HER2-60 (*n* = 22), and HER2-90 (*n* = 13) cells (231-Luc vs. HER2-60; *p* < 0.001; 231-Luc vs. HER2-90; *p* = 0.34).

To examine whether HER2 heterogeneity affects the malignant potential of breast cancer, we injected 231-Luc (triple negative cell line) and 2 HER2-positive cell lines, HER2-60 and HER2-90 into nude mice. At 3 weeks after injection, distant metastases were found in 21 out of the 24 mice (87.5%) injected with 231-Luc cells, 22 out of the 22 mice (100%) injected with HER2-60 cells, and in 9 out of the 13 mice (69.2%) injected with HER2-90 cells. Metastatic lesions were evident on bioluminescence imaging of the mice injected with HER2-60 cells at 21 days after intracardiac injection (Figure 1c,d). However, no metastasis developed in the mice injected with 231-Luc cells and a small number of metastasis was found in 1 out of 2 mice injected with HER2-90 cells at 21 days (Figure 1a,b,e,f). As shown in the representative bioluminescence imaging, mice injected with HER2-60 cells developed metastatic lesions earlier than mice injected with 231-Luc or HER2-90 cells (Figure 1a–f). The median survival days of mice injected with 231-Luc, HER2-60, and HER2-90 cells were 29 (*n* = 24), 24 (*n* = 22) and 30 (*n* = 13) days, respectively (Figure 1g). The mice injected with HER2-60 cells showed a significantly shorter survival time than the mice injected with 231-Luc and HER2-90 cells (*p* < 0.001). All the data of the survival days of mice are described in the supplementary data (Table S1). All the mice were investigated for brain, lung, liver, and bone (spine, legs) metastases in the H&E-stained specimens. Most of the metastasized tumors were found in the brain and bone, but a few were found in the lungs, but not in the liver. Brain metastases were present in all the representative mice as shown in the H&E-stained specimen (Figure 1a–f). The volume of brain metastasis was larger in the mice injected with HER2-60 cells than the mice injected with HER2-90 cells.

#### 2.1.2. HER2-60 Brain Metastasis Showed Low Expression of Caspase-1 on RNA Sequence Analysis

RNA sequence analysis was performed for metastatic brain tumors with surrounding mouse brain tissue [10]. Each read was distinguished as belonging to either human or mice. A total of 26,932 genes were identified in human cancer cells and 24,527 genes in mouse stromal cells. To compare the mRNA expression of the metastatic HER2-60 and HER2-90 cancer cells, 131 genes were upregulated in HER2-60 metastasized brain tumor cells with more than a 5-fold change and 108 genes were downregulated with less than a 5-fold change. Notably, 27 genes were upregulated and 38 genes were downregulated in the mouse stromal cells. All the gene lists are described in the supplementary data (Tables S2–S5). Tables S2 and S3 show the gene lists in cancer cells. Table S2 shows the upregulated genes in HER2-60 cells compared with the HER2-90 cells, and Table S3 shows the downregulated genes. Tables S4 and S5 show the gene lists in mouse stromal cells. Table S4 shows the upregulated genes in mouse stromal cells in the metastatic brain with HER2-60 cells compared with the metastatic brain with HER2-90 cells, and Table S5 shows the downregulated genes.

**Figure 2:**
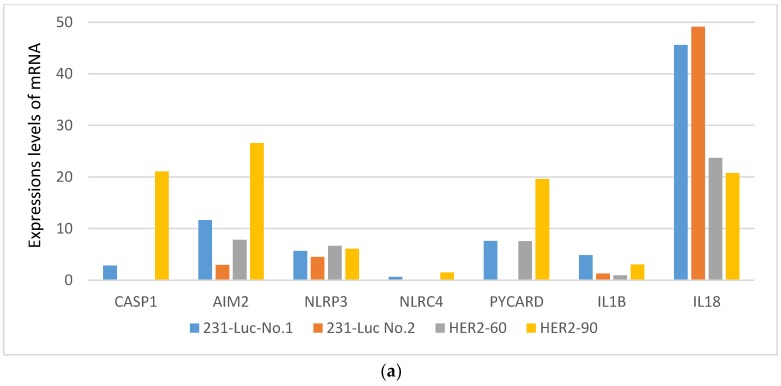

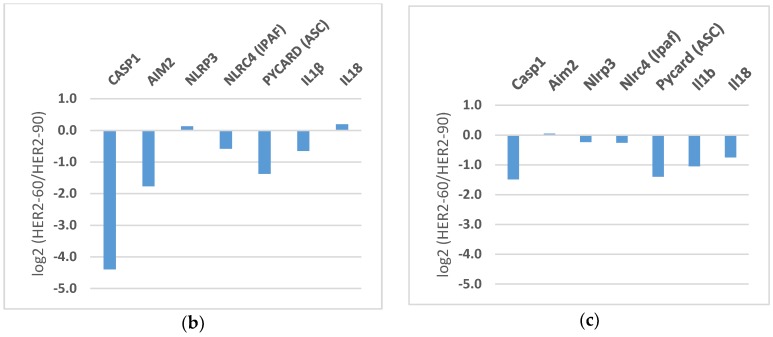
The expression levels of caspase-1 and its related genes were decreased in the metastatic brain with HER2-60 cells. (**a**) mRNA levels of caspase-1-related genes of cancer cells in the metastatic brains with 231-Luc, HER2-60, and HER2-90 cells. The values are shown in Table 1; (**b**,**c**) relative mRNA levels in the metastatic brains with HER2-60 cells compared with those with HER2-90 cells; (**b**) relative mRNA levels of cancer cells; (**c**) relative mRNA levels of mouse stromal cells. The values are shown in Table S6.

Among the genes in the cancer cells, *CASP-1* was downregulated in the HER2-60 brain metastasis more than 21 times than in the HER2-90 brain metastasis (Figure 2a, Table 1). Caspase-1 activation induces cell death called pyroptosis, which was identified as one of the programmed cell death mechanisms in macrophages infected by bacteria [11]. Caspase-1 induces the maturation and secretion of IL-1β and IL-18 [12]. These cytokines induce multiprotein complexes termed ‘inflammasomes’ such as nucleotide oligomerization domain (NOD)-like receptors (NLRs) and absent in melanoma (AIM2) [12]. NLR family pyrin domain-containing protein 3 (NLRP3) and NLR family caspase activation and recruitment domain (CARD) domain-containing protein 4 (NLRC4) are essential. *PYCARD* encodes an adaptor protein with caspase-1, which is composed of Pyrin domein and CARD [13].

To examine the expression levels of these caspase-1-related genes, the mRNA expression levels of *NLRP3*, *NLRC4*, *AIM2*, *PYCARD*, *IL-1β*, and *IL-18* were compared in the metastatic brains with 231-Luc, HER2-60, and HER2-90 cells. The expression levels of caspase-1-related genes except *NLRP3* and *IL-18* were downregulated in the HER2-60 cells compared with the HER2-90 cells. (Figure 2a,b). In mouse stromal cells, caspase-1-related genes except *NLRP3* and *AIM2* were downregulated in the HER2-60 cells (Figure 2c). Then we examined the expression levels of *CASP1* and *AIM2* by real time RT-PCR in mouse metastatic brains obtained using the same experimental procedure. The amounts of mRNA for *CASP1* and *AIM2* were decreased in HER2-60 cells compared with HER2-90 cells (Figure S1). These results were consistent with the results of the RNA sequence analysis.

We investigated the association of the expression of caspase-1 in the primary tumor and the prognosis of patients with breast cancer in a clinical database, cBioPortal. In 924 patients (800: stage 2, 115: stage 3, 9: stage 4), the low expression of caspase-1 was correlated with a poor prognosis (Cox regression coefficient = −0.10002, *p* = 0.014).

### 2.2. Clinical Study

HER2 Heterogeneous Expression Is Correlated with Resistance to Chemotherapy Plus Trastuzumab and Poor Prognosis in Patients with HER2-Positive Breast Cancer.

**Figure 3:**
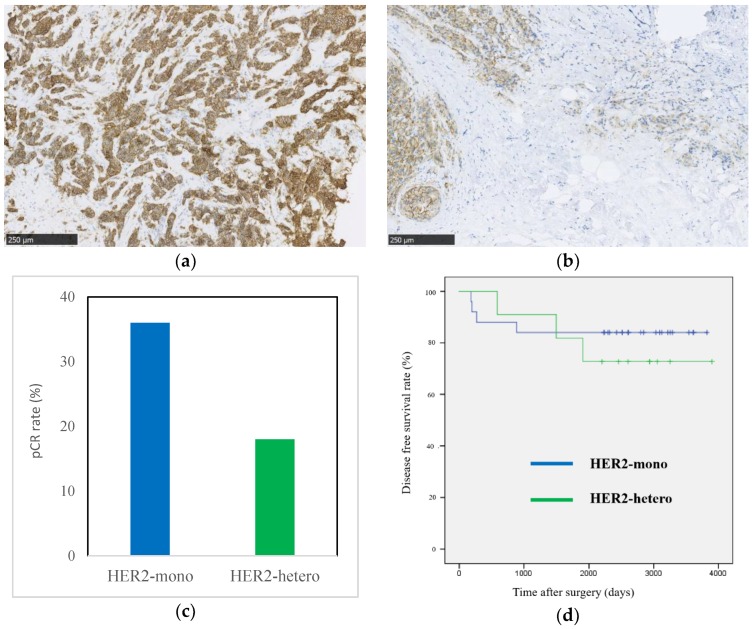
Heterogeneous HER2 expression is correlated with a high risk of relapse and resistance to chemotherapy plus trastuzumab in patients with HER2-positive breast cancer. (**a**) Representative IHC staining of HER2-mono tumors. Scale bar, 250 μm; (**b**) representative IHC staining of HER2-hetero tumors. Scale bar, 250 μm; (**c**) PCR rate of HER2-positive breast cancer patients treated with chemotherapy plus trastuzumab (*p* = 0.29); (**d**) Disease free survival (DFS) curves of patients with HER2-positive breast cancer after neoadjuvant chemotherapy plus surgery. The median DFS has not been reached (*p* = 0.97).

From the definition of HER2 heterogeneity by the immunohistochemistry (IHC) staining pattern, 25 cases and 11 cases were categorized into HER2-monotonous cases (HER2-mono) and HER2-heterogeneous cases (HER2-hetero), respectively. Heterogeneous HER2 expression was found in 56% of the IHC (2+) cases and in only 6% of the IHC (3+) cases. Representative patterns of HER2-mono and HER2-hetero are shown in Figure 3a,b, respectively.

The pathological complete response (pCR) rate was 36% (9/25) in HER2-mono cases and 18% (2/11) in HER2-hetero cases (Figure 3c, *p* = 0.29). At the median follow-up of 61 months, recurrent diseases were found more frequently in HER2-hetero cases (27%: 3/11) than in HER2-mono cases (16%: 4/25) (*p* = 0.51). Patients with HER2-hetero tumors tended to show a shorter disease-free survival (DFS) than patients with HER2-mono tumors (Figure 3d, *p* = 0.97).

## 3. Discussion

HER2 intratumoral heterogeneity is not usually evaluated in clinical practice. The fluorescence in situ hybridization (FISH) test in multiple regions may not be practical, although the ASCO/CAP guideline recommends that tumors equivocal for HER2, IHC (2+) or FISH score: 1.8–2.2 should be assessed by FISH in at least 2 representative fields of the invasive tumor [14]. This implies that it is worth examining HER2 genetic heterogeneity. Genetic heterogeneity of HER2 has been associated with breast cancers with low-grade HER2 amplification (FISH score: >2.2 and <4.0) or equivocal cases including IHC (2+) HER2 expression [6,15]. In the present study, heterogeneous HER2 expression was found in 56% of the IHC (2+) cases and in only 6% of the IHC (3+) cases. Our results are not consistent with past reports on the heterogeneity of HER2 expression associated with breast cancer with IHC (2+) equivocal cases.

From previous reports, intratumoral heterogeneity was associated with the progression and metastasis of breast cancer [4,5]. There are a few studies that have examined the clinical impact of intratumoral HER2 heterogeneity on prognosis [6,8]. In our mouse model, heterogeneous HER2 expression was associated with a short survival in metastatic breast cancer. However, as our clinical study consisted of a small number of patients and the results were not statistically significant, further studies with a large number of patients are necessary to specifically clarify the underlying mechanism.

HER2 is also overexpressed in other types of cancer besides breast cancer. In patients with HER2-amplified esophageal adenocarcinomas, heterogeneous HER2 amplification was observed in 17%, and the presence of HER2 heterogeneity was independently associated with worse disease-specific survival and overall survival (OS) [16].

However, it remains unknown why HER2 heterogeneity is associated with malignant potential. Molecular profiling classified breast cancer into 6 intrinsic subtypes. Estrogen receptor/progesterone receptor/HER2 positivity on IHC staining is thought to surrogate the intrinsic subtypes. However, different intrinsic subtypes are mixed in HER2-positive breast cancer, that is, 51% showed HER2-enriched type, 28% showed luminal type, and 21% showed the typical intrinsic subtypes for triple negative breast cancer such as basal type, claudin-low type, and normal-like type [17]. This suggests that the triple negative features are present in some HER2-positive breast cancers, which may be associated with the malignant potential. In the present study, mice injected with heterogeneous-HER2 cells (HER2-60) showed a shorter survival than mice injected with triple negative cells (231-Luc). Taken together, the interaction between HER2-positive cells and HER2-negative cells appear to accelerate the malignant potential.

The RNA sequence data demonstrated that CASP-1 and its related genes were more downregulated in the HER2-60 metastasis in the brain than in the HER2-90 metastasis, which indicates that the caspase-1-dependent pathway was suppressed in HER2-60 cells. A decreased level of caspase-1 protein expression was reported in primary prostate cancer compared with normal prostate tissues [18]. Casp1-/- mice reportedly showed increased tumorigenesis in colorectal cancer [19]. Loss of caspase-1 in gene expression was associated with worse survival in patients with gastric cancer [20]. Recently, it has been reported that caspase-1 mRNA expression was decreased in breast cancer tissues compared with tumor-adjacent normal tissues. It has also been shown that the proliferation and invasion abilities were increased by a caspase-1 small molecule inhibitor in MDA-MB 231 cells [21]. Increasing evidence suggest that caspase-1-dependent cell death (i.e., pyroptosis) contributes to tumor suppression in various cancers. The clinical database cBioPortal showed a correlation between the low expression of caspase-1 in the primary tumor and the poor survival of patients with breast cancer. These data also support our data.

The activation of inflammasomes has been reported to be associated with neurological diseases such as meningitis, stroke, and Alzheimer’s disease [13]. NLRP3 inflammasome is activated in microglia or macrophages in the central nervous system in case of meningitis or encephalitis [22]. NLRC4 inflammasome is also activated in microglia or macrophages in case of meningitis or encephalitis [23]. Previous reports have shown that the activation of inflammasomes is present in the central nervous system, which support our data that caspase-1-dependent pathway is activated in the brain under certain circumstances and may regulate metastasis of breast cancer into the brain.

## 4. Materials and Methods

### 4.1. Basic Study

#### 4.1.1. Cell Culture and Establishment of HER2-60 and HER2-90 Cell Lines

MDA-MB-231-Luc-D3H2LN (231-Luc) cells were obtained from Caliper Life Sciences (Perkin-Elmer, Waltham, MA, USA). The Methods for cell culture and establishment of the HER2-60 and HER2-90 cell lines were described previously [9]. Briefly, the HER2-60 cell line was generated from 231-Luc by introducing *HER2* wild type plasmid and sorting of HER2-overexpressing cells by neomycin (G418, 1200 μg/mL) selection and cell sorting. The HER2-90 cell line was produced from the HER2-60 cell line by repeated cell sorting of HER2-overexpressing cells.

#### 4.1.2. Brain Metastasis Model

Phosphate buffered saline (PBS), HER2-60 cells, or HER2-90 cells (1 × 10^5^ cells in 100 µL of PBS) were injected as a single-cell suspension into the left ventricle of 4-week-old female *Balb/c nu/nu* immune-deficient mice (Charles River, Burlington, MA, USA) that had been anesthetized by exposure to 1% to 3% isoflurane or intraperitoneal administration of somnopentyl (Kyoritsu Seiyaku, Tokyo, Japan).

#### 4.1.3. Bioluminescence Imaging

Mice anesthetized as described above were injected intraperitoneally with d-luciferin (20 mg/kg) at 10 to 20 min before whole-body imaging with an in vivo fluorescence imager (ClairvivoOPT; Shimadzu, Kyoto, Japan). The luminescence from injected cells starts appearing 2 weeks after the tumor cell injection.

#### 4.1.4. Selecting Samples for RNA Sequence Analysis

The brain was removed from the skull of each anesthetized mice and cut into 8 pieces using Brain Matrices (ASI Instruments, Warren, MI, USA). Of these 8 pieces, 4 pieces were preserved immediately in TRIzol reagent (Life Technologies, Carlsbad, CA, USA), and the other 4 pieces were preserved in formalin for IHC. Total RNA was isolated from the 4 brain pieces of each mouse and evaluated by determining the RNA integrity number. Real-time RT-PCR analysis was performed using specific primers for either mouse or human to optimally select a sample which has metastasized cancer cells. The real time RT-PCR primer sequences were described in 4.1.6.

#### 4.1.5. RNA Sequence

The mRNA libraries were prepared according to the TruSeq RNA Sample Prep Kit protocol and sequenced using a next-generation sequencer, Genome Analyzer IIx (Illumina, San Diego, CA, USA). RNA-seq reads were mapped to all RefSeq transcripts of human (hg19 coordinates) and mouse (mm10 coordinates) using Bowtie software [24]. Then, mouse and human reads were separated, and the gene expression profiles of each species were constructed using CASTIN software [25]. All expression values below 1 were set to 1 for fold change calculation to avoid extreme values.

For sample selection, we selected 2 samples of mice injected with PBS as normal control and 2 samples as metastatic brains with 231-Luc. The data obtained from the 2 samples with 231-Luc were very similar, thus we performed the next sequence with 1 sample for each HER2-60 and HER2-90.

#### 4.1.6. Real Time RT-PCR

The real-time RT-PCR protocol was described previously [10]. The real time RT-PCR primer sequences were as Table 2.

### 4.2. Clinical Study

#### 4.2.1. Definition of HER2 Genetic Heterogeneity

HER2 IHC staining was performed and assessed according to the ASCO/CAP HER2 test guideline recommendations. HER2 (2+) cases detected by IHC were also detected by fluorescence in situ hybridization (FISH). The ASCO/CAP guideline defined HER2 genetic heterogeneity as the presence of tumor cells with HER2/CEP 17 signal ratios higher than 2.2 in 5–50% of the tumor cells [14].

#### 4.2.2. Classification of HER2-positive Breast Cancer with or without HER2 Heterogeneity by IHC Staining

There were 36 patients with HER2-positive breast cancer who received neoadjuvant chemotherapy with trastuzumab and underwent surgery between January 2004 and December 2010. Tumors were divided into 2 types according to their HER2 staining pattern, that is, completely and partially (10–50%) stained cases. We defined these cases as HER2-monotonous tumors (HER2-mono) and HER2-heterogeneous tumors (HER2-hetero), respectively.

### 4.3. Survival Analysis

All survival data were plotted on Kaplan–Meier’s survival curves using SPSS software (version 24, Armonk, NY, USA). The log-rank test was used to compare the survival rates between the 2 groups. A *p*-value of <0.05 was considered to indicate a statistically significant difference.

For the mouse model, OS was defined as the time from injections of cancer cells into mice to death caused by breast cancer metastasis. Mice that died because of anesthesia or intracardiac injection complications were excluded from the survival analysis. For the clinical study, DFS was defined as the time from surgery to breast cancer relapse in distant organs.

In the clinical database, an overall survival analysis was conducted using the “survival” package in R (version 3.4.2, Vienna, Austria). Cox regressions were performed to evaluate the prognostic value of the CASP1 gene expression levels (z-scores) after controlling for tumor stage in the METABRIC dataset [26]. The gene expression levels of CASP1 and the clinical information in the METABRIC dataset were downloaded from cBioPortal [27]. Survival analysis was performed in 924 patients after excluding patients with sarcoma or phyllodes tumor, and tumor stage unknown, 0 or 1. The *p*-values for outcome correlation were calculated using the Wald test.

### 4.4. Ethics Statement

All experiments with mice were approved by the Ethics Committee of Keio University (ethical approval code: 08100, 8 May 2014). The clinical study was approved by the Ethics Committee of Tokyo Medical University (ethical approval code: 3638, 3 April 2017). Clinical data were managed using anonymous numerical codes.

## 5. Conclusions

We demonstrated that the heterogeneity of HER2 expression accelerated the development of metastases which caused the poor survival of mice with heterogeneous HER2 expression (HER2-60)*.* Caspase-1 and its related genes showed a lower expression in the metastatic brain with HER2-60 than with HER2-90. Further research with breast cancer tissue from a larger number of patients is required to reproduce the correlation of HER2-heterogeneity and poor survival. The low expression of caspase-1 could be a prognostic marker for early relapse, which will be investigated with patient samples in the near future. It is intriguing and worthwhile to further investigate this mechanism in future studies including the interaction between HER2-positive cells and HER2-negative cells, as well as that of cancer cells and stromal cells to definitively elucidate the mechanism underlying the possible association between HER2 heterogeneity and the malignant potential of HER2-positive breast cancer.

## Figures and Tables

**Table 1:** Expressions levels of mRNA.

Gene Symbol	231-Luc No. 1	231-Luc No. 2	HER2-60	HER2-90
*CASP1*	2.83	0	0	21.07
*AIM2*	11.62	2.96	7.80	26.59
*NLRP3*	5.65	4.51	6.67	6.10
*NLRC4*	0.61	0	0	1.49
*PYCARD*	7.59	0	7.56	19.61
*IL1B*	4.85	1.28	0.95	3.06
*IL18*	45.61	49.12	23.71	20.75

*CASP1: caspase-1*; *AIM2:absent in melanoma*; *NLRP3*: NLR family pyrin domain-containing protein 3; NLRC4: NLR family CARD domain-containing protein 4; PYCARD: Apoptosis-associated speck-like protein containing a CARD; IL1B: interleukin 1 beta; IL-18: interleukin 18.

**Table 2:** Primer sequences for real time RT-PCR.

Primer	Forward	Reverse
mouse *Gapdh*	5-ACTAACATCAAATGGGGTGAGGCC-3	5-GGATGCATTGCTGACAATCTTGAGTGA-3
human *GAPDH*	5-CAAAATCAAGTGGGGCGATGCTGGC-3	5-GGCATTGCTGATGATCTTGAGGCT-3
*CASP1*	5-TCCCTAGAAGAAGCTCAAAGGATATG-3	5-CGTGTGCGGCTTGACTTG-3
*AIM2*	5-CAGAAATGATGTCGCAAAGCA-3	5-TCAGTACCATAACTGGCAAACAG-3

## Supplementary Materials

Supplementary materials can be found at http://www.mdpi.com/1422-0067/19/8/2158/s1.

## Abbreviations

HER2	human epidermal growth factor receptor 2
CEP17	centromere on chromosome 17
NLRs	nucleotide oligomerization domain (NOD)-like receptors
AIM2	absent in melanoma
NLRP3	NLR family pyrin domain-containing protein 3
NLRC4	NLR family caspase activation and recruitment domain (CARD) domain-containing protein 4
PYCARD	apoptosis-associated speck-like protein containing a CARD
IHC	immunohistochemistry
DFS	disease free survival
ASCO/CAP	American society of clinical oncology and College of American pathologist
OS	overall survival
