# Supplementary figures and images for: HER2 Heterogeneity Is Associated with Poor Survival in HER2-Positive Breast Cancer
Source: Int J Mol Sci. 2018 Jul 24;19(8):2158. doi: 10.3390/ijms19082158 (PMC6121890; doi:10.3390/ijms19082158)

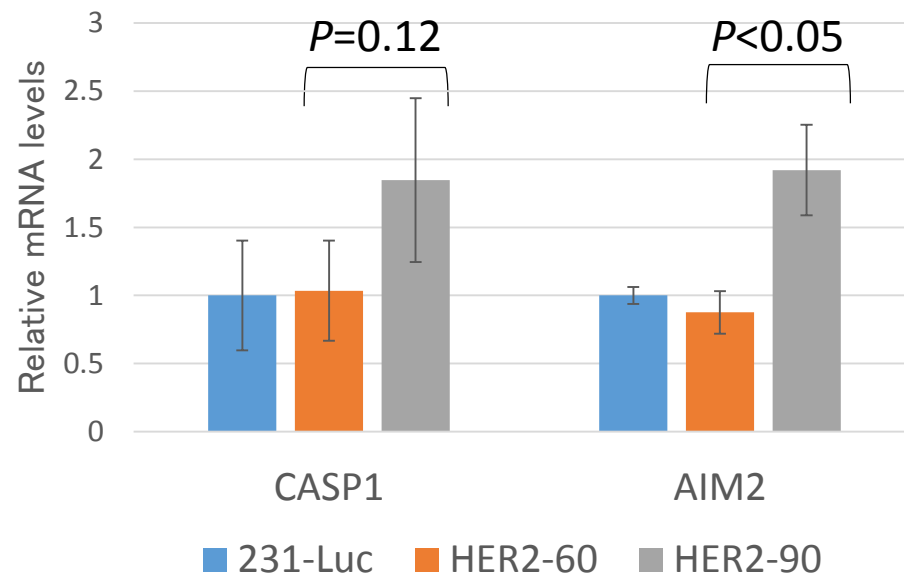

Supplement: Supplementary file 1 [file ijms-19-02158-s001.zip › Supplementary figure S1 Hosonaga.pdf]
